# Supplementary material for: A machine learning approach for identification of gastrointestinal predictors for the risk of COVID-19 related hospitalization
Source: PeerJ. 2022 Mar 21;10:e13124. doi: 10.7717/peerj.13124 (PMC8944335; doi:10.7717/peerj.13124)
Supplement: Supplemental Information 5 [file peerj-10-13124-s005.docx]

Name:

State identification number:

Telephone number:

=====================================================================

1. Reason for PCR /antigen test:

a. Symptoms (please specify):

■ Fever

■ Cough

■ Weakness

■ Rhinitis

b. Contact with the positive

c. Other:

2. Do you use proton pump inhibitors for a long period of time? (medicines to affect the acid in the stomach, such as Helicide, Nolpaza, Pantomyl, Lanzul and such)

Yes No

3. Do you have the following symptoms for a long period of time?

a. I have no long-term digestive problems

b. Diarrhea

c. Constipation

d. Stomachache

e. Bloating

f. Feeling sick (vomiting)

g. Heartburn

h. Other (digestive related):

4. Have any of the above symptoms worsened in the past 3-5 days?

Yes No

5. Have you had any of these symptoms in the past 3-5 days?

i. Diarrhea

j. Constipation

k. Stomachache

l. Bloating

m. Feeling sick (vomiting)

n. Heartburn

o. Other (digestive related):

Notes or any, more detailed information:
