# Supplementary material for: A machine learning approach for identification of gastrointestinal predictors for the risk of COVID-19 related hospitalization
Source: PeerJ. 2022 Mar 21;10:e13124. doi: 10.7717/peerj.13124 (PMC8944335; doi:10.7717/peerj.13124)
Supplement: Supplemental Information 6 [file peerj-10-13124-s006.docx]

Meno:

Rodné číslo:

Telefónne číslo:

===========================================================================

1. Dôvod PCR/antigénového vyšetrenia:
   1. Príznaky (prosím konkretizujte):
      - Zvýšená teplota
      - Kašeľ
      - Slabosť
      - Nádcha
   2. Kontakt s pozitívnym
   3. Iné:

2. Užívate dlhodobo inhibítory protónovej pumpy? (lieky na ovplyvnenie kyseliny v žalúdku, ako napríklad Helicid, Nolpaza, Pantomyl, Lanzul a podobne)

Áno Nie

3. Sú u Vás prítomné dlhodobo nasledovné príznaky?

- 1. Nemám žiadne dlhodobé ťažkosti s trávením
  2. Hnačka
  3. Zápcha
  4. Bolesti brucha
  5. Nafukovanie
  6. Pocity na zvracanie
  7. Pálenie záhy
  8. Iné (súvisiace s trávením):

4. Zhoršili sa u Vás niektoré z hore uvedených príznakov v priebehu uplynulých 3-5 dní?

Áno Nie

5. Objavili sa u Vás niektoré z uvedených príznakov v priebehu uplynulých 3-5 dní?

- 1. Hnačka
  2. Zápcha
  3. Bolesti brucha
  4. Nafukovanie
  5. Pocity na zvracanie
  6. Pálenie záhy
  7. Iné (súvisiace s trávením):

Poznámka alebo ľubovoľné, upresňujúce informácie:
